# Fatality risk and issues of inequity among vulnerable road users in South Africa

**DOI:** 10.1371/journal.pone.0261182

**Published:** 2021-12-31

**Authors:** Anesh Sukhai, Rajen Govender, Ashley van Niekerk

**Affiliations:** 1 Institute for Social and Health Sciences, University of South Africa, Johannesburg, South Africa; 2 Masculinity and Health Research Unit, University of South Africa and South African Medical Research Council, Cape Town, South Africa; Shenzhen University, CHINA

## Abstract

**Background:**

Contextual effects from the physical and social environment contribute to inequitable protection for a large proportion of road users, especially in low- and middle-income countries like South Africa where distorted urban planning and socio-spatial disparities from the apartheid era prevail.

**Objectives:**

This paper examines the differentiated risk of road traffic crashes and injuries to vulnerable road users in South Africa, including pedestrians, females and users of some modes of public transport, in relation to characteristics of the crashes that proxy a range of contextual influences such as rurality and socio-economic deprivation.

**Methods:**

The study is based on a descriptive analysis of 33 659 fatal crashes that occurred in South Africa over a three-year period from 2016–2018. Measures of simple proportion, population-based fatality rate, “impact factor” and crash severity are compared between disaggregated groups using Chi-Square analysis, with the Cramer’s V statistic used to assess effect size.

**Results and significance:**

Key findings show a higher pedestrian risk in relation to public transport vehicles and area-level influences such as the nature of roads or extent of urbanity; higher passenger risk in relation to public transport vehicles and rurality; and higher risk for female road users in relation to public transport vehicles. The findings have implications for prioritising a range of deprivation-related structural effects. In addition, we present a “User-System-Context” conceptual framework that allows for a holistic approach to addressing vulnerability in the transport system. The findings provide an important avenue for addressing the persistently large burden of road traffic crashes and injuries in the country.

## Introduction

Factors relating to the physical and social environment are major contributors, along with the contributing effects relating to behavioural- and vehicle-related factors, to the large burden of road traffic crashes and injuries (RTCIs) in many low- and middle-income country (LMIC) settings [1–4]. The contextual effects from physical and socio-economic environmental disadvantage are experienced differentially as a range of “vulnerabilities” in the road traffic system, resulting in inequitable protection for a large proportion of road users [1–4].

### Burden of RTCIs

Of an estimated 1.35 million deaths that occurred worldwide in 2016, the highest fatality rates were found for LMICs and for the African region. The average rate for LMICs of 27.5 fatalities per 100 000 population was shown to be 3 times the rate of 8.3 for high-income countries, while the 26.6 rate for the African region was 1.5 times the average Global rate of 18.2 [5]. Vulnerable road users (VRUs), comprising pedestrians, cyclists, and motorised 2- and 3-wheelers are most affected (54% of global road traffic deaths) with the highest proportion of pedestrian deaths found for the African region (40%, and twice the average Global proportion of 23%) [5]. In addition, on average, nearly three (2.7) times more males than females die from road traffic injuries (RTIs) [6].

The fatality rate for South Africa in 2016 was estimated at 25.9 per 100 000 population [5]. Earlier research showed the fatality rate for males to be 3.5 times higher than that for females (34.9 vs. 10.1 per 100 000 population, respectively) [7]. Proportionally, males accounted for roughly three-quarters of road traffic deaths [8, 9] with the highest M:F ratios of 4.6 and 3.2 among young to middle-aged adults in the 20–39 year and 40-59-year age groups, respectively [9]. Like many other countries in Africa, pedestrian injury is the leading cause of road traffic death in South Africa, accounting for 38% of all road traffic deaths in 2018, followed by passenger and driver deaths (33% and 26%, respectively) [8]. In addition to the disproportionately large mortality burden with associated hospitalisations, disabilities and psychosocial trauma, RTCIs were shown to have a considerable impact on the South African economy with the cumulative cost estimated at R166 billion in 2018 [10], equating to 3.8% of the country’s Gross Domestic Product [11].

### Road user vulnerability from a demographic, biomechanical and capability perspective

The term “vulnerable road users” is generally applied to road users who are physically less protected than occupants within closed vehicles, and include pedestrians, cyclists and motorcyclists [12]. In addition, based on a combination of demographics, biomechanics and capabilities, some groups of the population may be even more vulnerable, including children, older persons and people living with disabilities [12, 13].

These vulnerable groups have received increasing attention and priority within global health and development agendas. For example, within the United Nations (UN) Sustainable Development Goals (SDGs), SDG target 11.2 provides for access to “safe, affordable, accessible and sustainable transport systems…with special attention to the needs of those in vulnerable situations, women, children, people with disabilities and older people” [14]. Equity, defined as the “absence of avoidable, unfair, or remediable differences among groups of people, whether those groups are defined socially, economically, demographically or geographically or by other means of stratification” [15], is also a fundamental principle and priority for achieving the objectives and targets within the UN SDG and the related Universal Health Coverage (UHC) Agendas [14, 16].

### Road user vulnerability in relation to the transport system and other contextual influences

The above vulnerable road user categorisations, while reflecting vulnerability from transport mode and human biomechanical perspectives, do not consider the vulnerability conferred to vulnerable road users by the traffic and social environments [1–4]. In a study of a wide range of traffic, social and other physical environmental effects in South Africa, the key predictors of the occurrence of RTCIs (along with the relatively direct effect of drink-driving) included contextual effects relating to rurality, socio-economic deprivation, travel exposure and measures of crime and violence [4]. An important category of contributing effects to the occurrence of RTCIs in general relates to deficiencies in the transportation system that arise from the prioritization of vehicle occupants and motorized transport at the expense of the safe mobility needs of vulnerable road users. Khayesi [17] highlights these effects as a reflection of “vulnerable transport planning” and argues for a shift in viewing vulnerability in the transport system from vulnerable road users to the factors located within transport planning and associated land use environment.

In addition to the dimensions of road user vulnerability arising from human biomechanics and vulnerable transport system perspectives, transport planning in South Africa is also characterised by inequitable provision for the infrastructural needs of vulnerable road users as a result of distorted urban planning and socio-spatial disparities perpetuated by the apartheid regime with ongoing consequences [18–20]. The World Health Organization (WHO) refers to these effects as “structural determinants” that “generate or reinforce social stratification in the society and that define individual socioeconomic position” as the preferred term over “distal factors” in order to emphasise the causal hierarchy of social determinants that contribute to health inequities [21]. Proximal factors generally refer to those factors that directly affect health while distal factors affect health indirectly.

While Khayesi [17] makes reference to other economic, social, technological and political factors that combine with transport planning decision-making in effecting vulnerable transport planning, an emphasis on related though structurally-induced effects, as in the South African setting, is important to underscore these as distinct for the purposes of remediation. This allows for disentangling and increasing the scope for collective efforts to shift focus from road user vulnerability (and blaming) to the physical traffic environment as well as the structural factors that underpin vulnerability among road users. These structural forces may also be regarded as distinct contributing effects. For example, in the case of pedestrians in disadvantaged settings, in addition to their physical or biomechanical limitations, they are vulnerable to the effects of both transport planning and other related decision-making processes, together with the vulnerability that is conferred from exposure to a wide range of socio-economic deprivations.

#### A User-System-Context hierarchical framework for vulnerability in the transport system

**Fig 1:**
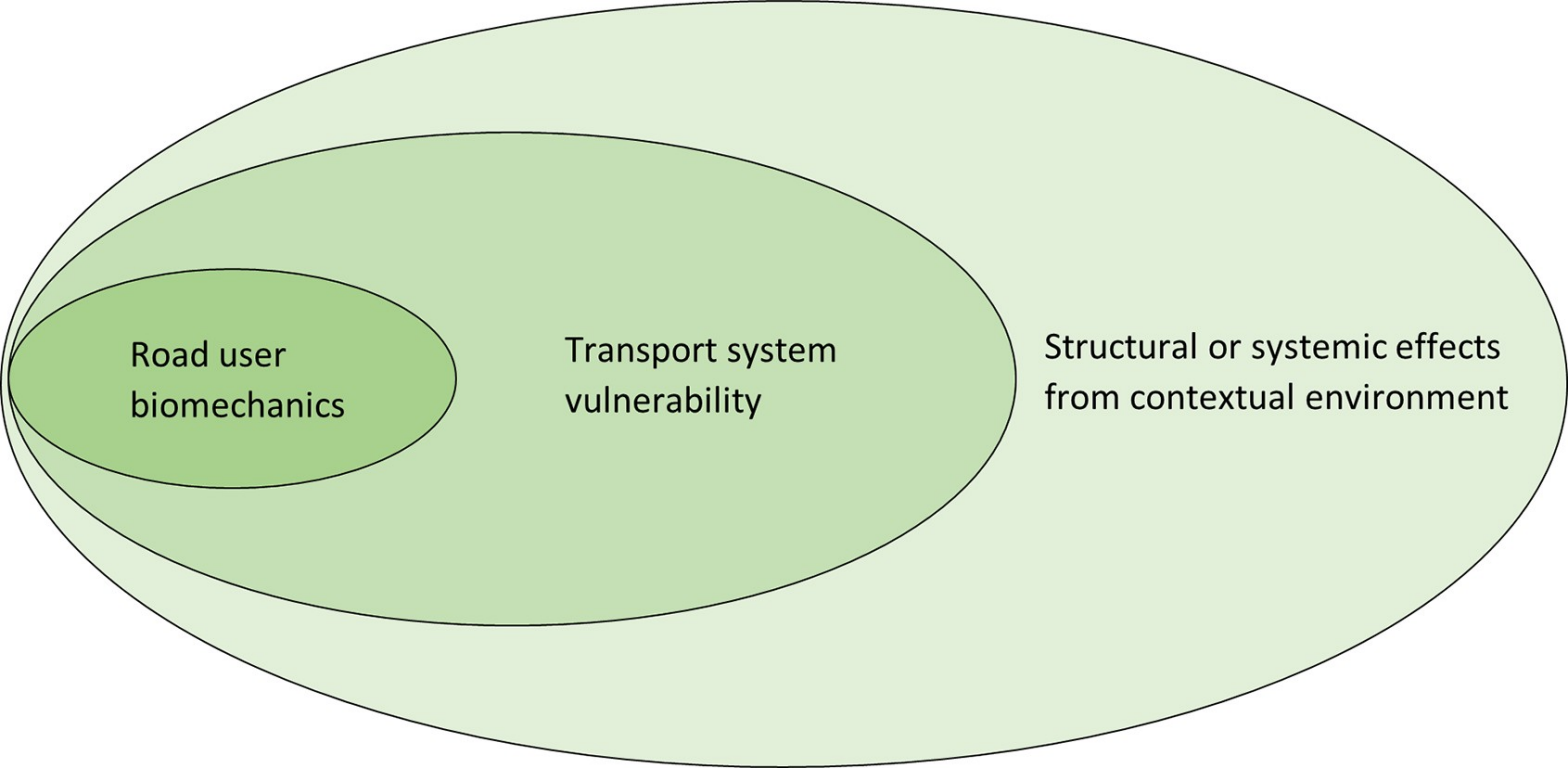
Nested effects pertaining to road users, the transport system and the contextual environment on a proximal to distal continuum.

Following from above, for the South African context we propose a tri-dimensional consideration of vulnerability in road users: the first two dimensions relate to human biomechanics and vulnerable transport systems, and the third relates to influences of systematic social and economic disadvantage between areas and population groups. We further suggest that, from a risk and intervention perspective, it is instructive to examine this 3-dimension “User-System-Context” as a nested or hierarchical framework for vulnerability in the transport system that may be considered on a proximal-to-distal causality continuum with road user biomechanics being at the proximal end and contextual effects relating to deprivation being at the distal end (see Fig 1 below). Within such a continuum, the distinctiveness of contextual influences becomes further apparent whereby vulnerability within the transport system from prevailing socio-economic-political forces may be considered as being underpinned by other contextual and historical forces that confers a systematic or entrenched disadvantage and risk. We also suggest that females be considered within the categorisation of vulnerable road users (from a human biomechanics perspective) as well as passengers of public transport (from their significant vulnerability relating to contextual experiences of unsafety). Below we review some of the key literature for the South African setting pertaining to vulnerability in relation to these proposed additional vulnerable road users, along with that of pedestrians.

#### Pedestrian and public transport passenger vulnerability

As indicated, pedestrians followed by passengers account for the largest proportion of road traffic deaths in South Africa [8]. The considerable burden of pedestrian and public transport passenger fatalities is largely underpinned by the ongoing impact of apartheid urban planning and socio-economic policies that legislated that especially black African communities be located great distances away from key economic nodes in the country [18–20]. The term black African (and other racial identities including coloured, Indian, and white) refer strictly to demographic markers which are important to measure and understand the legacy effects of group based structural deprivation perpetrated on local communities by the previous apartheid regime, and are not intended to denote any inherent group or individual characteristics. The travel over large distances to and from work and other services rendered (and continue to render) such communities at great disadvantage through heightened risk and vulnerability to RTCIs. For example, according to the 2020 National Household Travel Survey (NHTS), more than one-third (35%) of the population had to rely on public transport for their daily commute, with average times spent traveling to work being 107 mins for train travel, 84mins for bus travel and 63 mins for taxi travel [22]. Further, while pedestrians in deprived international settings have been shown to incur greater traffic-related risk exposure from greater mobility [23] or from needing to cross roads more often [24], other important considerations in marginalised settings in South Africa may relate to a general lack of safe pedestrian-related infrastructure such as for the crossing of roads or for the physical separation of vulnerable road users from motorised traffic [19, 20].

In the case of public transport, additional risk is conferred to passengers, especially in disadvantaged settings, who need to use generally unsafe public transport, as is the case with the large-scale and unregulated minibus taxi industry [20, 25]. The general risks with informal public transport, especially minibuses, may relate to a range of factors including passenger overloading, high risk driving behaviours, unrestrained passengers, and driver fatigue [25]. Public transport also includes the added exposure from pedestrian travel at both ends of the public transport journey, which can be excessive in the South African setting. For example, based on an analysis of national travel survey data, walking times to the nearest taxi stop were calculated at up to 49 mins (average 13 mins), and up to 78mins (average 13 mins) to the nearest bus stop. The combined walking time at both ends of the public transport journey for work-related trips was up to 52 mins (average 13 mins) [4].

#### Female vulnerability

A particular issue with the vulnerability of pedestrians and public transport users is the added vulnerability experienced by female road users. Further to the increasing recognition and prioritization of women within Global health and development agendas such as within the UN SDG and UHC Agendas [14, 16], there is also a growing body of research that in recent years highlight the experiences of vulnerability by females in the road traffic environment as well as their added risk to RTCIs [26–28]. In many African countries, females are reported to experience relatively greater risk exposure than men from walking and public transport, with these differences being more pronounced in rural areas [1, 29, 30].

In South Africa, females are generally more likely than males to use public transport, with the exception for train travel [26]. In 2013, the most commonly used mode of transport for day travel for females was as a taxi passenger (49.5% versus 43.4% for males), with higher female-male differentials found for generally disadvantaged black African (59.9 female vs. 53.2% male) and Coloured (27.3 female vs. 23.3 male) communities [26]. In addition, with using trains, heads of households expressed particular safety concerns regarding the walk to and from the station (56.6%), the time spent on the train (47.3%) and the time spent at train stations (32.4%), being safety issues expected to convey relatively greater vulnerability to females than males [27, 31]. In terms of the purpose of trips, female-male disparities were most apparent for trips undertaken for health or medical purposes (either for themselves or with caring for others) and found more especially for black African females (3.9%) versus black African males (2.2%) [26]. While the relatively higher care burden on women reflects on patriarchal social norms [26], poor transport and infrastructure along with experiences of unsafety are clearly impediments to this important role.

The use of mobility or travel exposure metrics such as time or distance of travel are also helpful in quantifying the differentiated risks experienced by females and male road users [28, 32] whereby road user travel is adjusted for the amount of travel usage and hence the volume of accident opportunities that a road user encounters [33, 34]. Whilst research using such exposure-based indicators are not available for the South African context, international research has shown a general preponderance of males in RTCIs including higher exposure-based fatality rates for both male drivers and male pedestrians [6]. Based on the risk conferred by a particular road user on other road users, however, men were shown to pose a higher per-km risk than women to other road users for all modes of transport with exception for buses [28].

In this paper we examine vulnerability in the transport environment in South Africa using the proposed nested 3-dimension “User-System-Context” proximal-to-distal causality continuum framework, thereby contributing to the growing body of research pertaining to road user risk and associated issues of vulnerability and inequity in the South African road traffic environment. We concentrate on the differentiated risk to pedestrians (vs vehicle occupants) and to females (vs. males) in relation to factors pertaining to the characteristics of the crashes, including the involvement of public transport vehicles. These characteristics, deemed as likely to differentiate risk among these vulnerable road users, also provide proxy indications of contextual influences such as rurality and socio-economic deprivation. This analysis, using the combination of road user groupings and crash characteristics provides for initial research with adopting the proposed conceptual framework along with preliminary perspectives (based on the available data) on road user vulnerability from an equity and social justice perspective.

## Methods

### Data on road traffic fatalities

The data on RTCIs derives from crash investigation data routinely collected by the Road Traffic Management Corporation (RTMC), a national statutory entity serving as the lead agency for road safety in the country. The RTMC is mandated by Act of Parliament to administer a national database of fatal road traffic crashes, stipulated in the RTMC Act, Act No 20 of 1999. The RTMC thus, as part of its statutory mandate, routinely collects and maintains data on all road traffic crashes in the country, including those for which there are one or more fatalities. This study’s data was obtained through a formal agreement on collaborative research with the RTMC. Ethical approval was not required for this research, based on provisions in the South African National Department of Health’s “Ethics in Health Research Principles, Processes and Structures” that provides oversight to ethical considerations in health research undertaken in the country [35]. Specifically, the guidelines indicate that “Research that relies exclusively on publicly available information or accessible through legislation or regulation usually need not undergo formal ethics review” (Section 1.1.8.), and “Research that relies exclusively on secondary use of anonymous information …… usually need not undergo formal ethics review, provided that no identifiable information is generated” (Section 1.1.10.) [35].

The RTMC data is based on routine collection of data on RTCIs, undertaken in partnership with South African Police Services (SAPS) using a standardised “Culpable Homicide Observation Form” (published elsewhere, see Govender et al [10]). Data collected includes key features of the crash, the vehicles involved, and the characteristics of victims where fatalities are involved. Whilst the RTMC data is the most comprehensive data on fatal RTCIs available nationally, it is not possible to further disaggregate passenger fatalities to reflect public transport users, nor is it possible to examine road user combinations such as a combined female-pedestrian grouping, although some limited inferences can be made from the disaggregated analyses.

### Sample

Cases analysed for this research comprised a total of 33 659 fatal crashes that occurred over a three-year period from 1 January 2016 to 31 December 2018. The cases included only fatal crashes for which the cause of the crash was assessed as being driver attributed, and for which there was information on specific driver behaviour attribution and road user details (transport mode and sex). The 33 659 fatal crashes involved 43 959 vehicles and resulted in 41 011 fatalities.

### Variables and statistical analyses

Disaggregated numbers of crashes and of total fatalities were used as outcome measures. The explanatory variables included the following hypothesized risk-related crash characteristic data available in the dataset: type of crash in terms of counterparts involved; type of vehicles involved; temporal effects including day, week, and vacation periods; and spatial effects including province and municipality of crash. The categories for the above risk-related variables are reflected in Tables 1–3, and the formulation and justification for the variable categories have been detailed in earlier research (see Govender et al [10]).

**Table 1:** Crash and temporal risk factors for fatal crashes by road user mode of transport.

	Driver		Passenger		Pedestrian		Pedestrian vs. vehicle occupant		
	n (%)	IF^	n (%)	IF^	n (%)	IF^	*P* *	Cramer’s V	Risk Ratio (CI)
**Vehicle type**									
Light vehicle	8509 (82.1%)	1.1	9868 (75.4%)	1.0	9098 (73.7%)	1.0	<0.01	0.05	0.94 (0.93–0.95)
Public transport	672 (6.5%)	0.5	2105 (16.1%)	1.3	2135 (17.3%)	1.4	<0.01	0.08	**1.46** (1.39–1.54)
Heavy vehicle	1005 (9.7%)	1.1	1080 (8.3%)	1.0	1034 (8.4%)	1.0	0.11	0.01	0.94 (0.88–1.01)
Motorcycle	184 (1.8%)	0.9	33 (0.3%)	0.1	72 (0.6%)	0.3	<0.01	0.02	0.63 (0.48–0.82)
**Day period**									
Night	6174 (57.9%)	1.1	7086 (52.9%)	1.0	8903 (56.7%)	1.0	<0.01	0.02	1.03 (1.01–1.05)
Day	4499 (42.2%)	0.9	6320 (47.1%)	1.1	6812 (43.4%)	1.0	<0.01	0.02	0.96 (0.94–0.99)
**Week period**									
Long Weekend	1284 (12.0%)	1.0	1945 (14.5%)	1.2	1875 (11.9%)	1.0	<0.01	0.02	0.89 (0.84–0.94)
Weekend	5553 (52.0%)	1.0	7009 (52.3%)	1.0	7758 (49.4%)	1.0	<0.01	0.03	0.95 (0.93–0.97)
Weekday	3836 (35.9%)	1.0	4452 (33.2%)	0.9	6082 (38.7%)	1.0	<0.01	0.04	1.12 (1.10–1.15)
**Vacation period**									
Non-Vacation	7607 (71.3%)	1.0	9146 (68.2%)	1.0	11313 (72.0%)	1.0	<0.01	0.03	1.04 (1.02–1.05)
Vacation	3066 (28.7%)	1.0	4260 (31.8%)	1.1	4402 (28.0%)	1.0	<0.01	0.03	0.92 (0.89–0.95)

* 2-tailed P-value, based on Yates corrected Chi-Square

^ Impact Factor

**Table 2:** Area-level risk factors for fatal crashes by road user mode of transport.

	Driver		Passenger		Pedestrian		Pedestrian vs. vehicle occupant		
	n (%)	IF^	n (%)	IF^	n (%)	IF^	*P* *	Cramer’s V	Risk Ratio (CI)
**Road surface type**									
Dirt	43 (0.4%)	0.7	45 (0.3%)	0.6	99 (0.6%)	1.1	<0.01	0.02	**1.73** (1.30–2.31)
Gravel	703 (6.6%)	1.0	1067 (8.0%)	1.2	706 (4.5%)	0.7	<0.01	0.06	0.61 (0.56–0.67)
Paved**	9877 (93.0%)	1.0	12251 (91.7%)	1.0	14770 (94.8%)	1.0	<0.01	0.05	1.03 (1.02–1.03)
**Road surface condition**									
Dry	9836 (93.6%)	1.0	12390 (93.6%)	1.0	14903 (96.0%)	1.0	<0.01	0.05	1.03 (1.02–1.03)
Wet	676 (6.4%)	1.3	851 (6.4%)	1.3	615 (4.0%)	0.8	<0.01	0.05	0.62 (0.56–0.68)
**Province-Municipality**									
EC	1214 (11.4%)	1.0	1875 (14.0%)	1.2	1831 (11.7%)	1.0	<0.01	0.02	0.91 (0.86–0.96)
Local	921 (8.6%)	1.0	**1581 (11.8%)**	1.3	1256 (8.0%)	0.9	<0.01	0.04	0.77 (0.72–0.82)
Metro	293 (2.8%)	0.9	294 (2.2%)	0.7	575 (3.7%)	1.2	<0.01	0.04	**1.50** (1.34–1.68)
FS	887 (8.3%)	1.3	1155 (8.6%)	1.3	752 (4.8%)	0.7	<0.01	0.07	0.56 (0.52–0.61)
Local	737 (6.9%)	1.3	971 (7.2%)	1.4	539 (3.4%)	0.7	<0.01	0.08	0.48 (0.44–0.53)
Metro	150 (1.4%)	1.0	184 (1.4%)	1.0	213 (1.4%)	1.0	0.83	0.00	0.98 (0.82–1.16)
GP	**2019 (18.9%)**	0.9	1823 (13.6%)	0.7	**3808 (24.2%)**	1.2	<0.01	**0.10**	1.52 (1.46–1.58)
Local	530 (5.0%)	1.1	493 (3.7%)	0.8	651 (4.1%)	1.0	0.63	0.00	0.98 (0.89–1.07)
Metro	1489 (14.0%)	0.8	1330 (9.9%)	0.6	3157 (20.1%)	1.2	<0.01	**0.12**	**1.72** (1.64–1.80)
KZN	1702 (16.0%)	0.8	**2268 (16.9%)**	0.8	**3790 (24.1%)**	1.2	<0.01	0.09	1.46 (1.41–1.52)
Local	1268 (11.9%)	0.9	**1823 (13.6%)**	1.1	**2113 (13.5%)**	1.0	0.08	0.01	1.05 (0.99–1.10)
Metro	434 (4.1%)	0.6	445 (3.3%)	0.5	1677 (10.7%)	**1.5**	<0.01	**0.14**	**2.92** (2.70–3.16)
LIM	1339 (12.6%)	1.1	2038 (15.2%)	**1.4**	1430 (9.1%)	0.8	<0.01	0.07	0.65 (0.61–0.69)
Local	1339 (12.6%)	1.1	2038 (15.2%)	1.4	1430 (9.1%)	0.8	<0.01	0.07	0.65 (0.61–0.69)
Metro	NA	NA	NA	NA	NA	NA	NA	NA	NA
MP	1404 (13.2%)	1.3	1667 (12.4%)	1.2	1289 (8.2%)	0.8	<0.01	0.07	0.64 (0.60–0.68)
Local	1404 (13.2%)	1.3	1667 (12.4%)	1.2	1289 (8.2%)	0.8	<0.01	0.07	0.64 (0.60–0.68)
Metro	NA	NA	NA	NA	NA	NA	NA	NA	NA
NC	336 (3.2%)	1.1	537 (4.0%)	**1.5**	294 (1.9%)	0.7	<0.01	0.05	0.52 (0.45–0.59)
Local	336 (3.2%)	1.1	537 (4.0%)	1.5	294 (1.9%)	0.7	<0.01	0.05	0.52 (0.45–0.59)
Metro	NA	NA	NA	NA	NA	NA	NA	NA	NA
NW	950 (8.9%)	1.2	1087 (8.1%)	1.1	947 (6.0%)	0.8	<0.01	0.05	0.71 (0.66–0.77)
Local	950 (8.9%)	1.2	1087 (8.1%)	1.1	947 (6.0%)	0.8	<0.01	0.05	0.71 (0.66–0.77)
Metro	NA	NA	NA	NA	NA	NA	NA	NA	NA
WC	822 (7.7%)	0.9	956 (7.1%)	0.8	1574 (10.0%)	1.1	<0.01	0.05	1.36 (1.27–1.45)
Local	483 (4.5%)	1.1	662 (4.9%)	1.2	586 (3.7%)	0.9	<0.01	0.02	0.78 (0.71–0.86)
Metro	339 (3.2%)	0.7	294 (2.2%)	0.5	**988 (6.3%)**	1.3	<0.01	0.09	**2.39** (2.17–2.64)
**Total Local**	7968 (74.7%)	1.1	10859 (81.0%)	1.2	9105 (57.9%)	0.9	<0.01	**0.22**	0.74 (0.73–0.75)
**Total Metro**	2705 (25.3%)	0.8	2547 (19.0%)	0.6	6610 (42.1%)	1.3	<0.01	**0.22**	1.93 (1.87–1.99)

* 2-tailed P-value, based on Yates corrected Chi-Square

** Includes tar and concrete surfaces

^ Impact Factor

# Abbreviations for provinces: EC = Eastern Cape; FS = Free State; GP = Gauteng; KZN = KwaZulu-Natal; LIM = Limpopo; MP = Mpumalanga; NC = Northern Cape; NW = North West; WC = Western Cape

**Table 3:** Crash and temporal risk factors for fatal crashes by road user sex.

	Male		Female		Male vs. female		
	n (%)	IF^	n (%)	IF^	*P* *	Cramer’s V	Risk Ratio (CI)
**Crash cause**							
Driver factor	13441 (47.0%)	1.1	4064 (48.3%)	1.1	0.03	0.01	1.03 (1.00–1.06)
Pedestrian factor	8859 (31.0%)	0.9	2617 (31.1%)	0.9	0.78	<0.01	1.01 (0.97–1.04)
Vehicle factor	1041 (3.6%)	1.0	449 (5.3%)	1.5	<0.01	0.04	**1.47** (1.32–1.64)
Road factor	1644 (5.8%)	1.0	501 (6.0%)	1.1	0.48	<0.01	1.04 (0.94–1.14)
Hit and run	3627 (12.7%)	0.9	776 (9.2%)	0.7	<0.01	0.05	0.73 (0.68–0.78)
**Crash type**							
Driver/Driver	7886 (30.1%)	1.2	2744 (33.0%)	1.4	<0.01	0.03	1.10 (1.06–1.14)
Driver/Pedestrian	9279 (35.4%)	0.8	2806 (33.8%)	0.8	0.01	0.02	0.95 (0.92–0.99)
Driver mainly	9019 (34.4%)	1.0	2765 (33.3%)	1.0	0.05	0.01	0.97 (0.93–1.00)
**Vehicle type**							
Light vehicle	21168 (76.4%)	1.0	6519 (75.1%)	1.0	0.02	0.01	0.98 (0.97–1.00)
Public transport	3389 (12.2%)	1.0	1555 (17.9%)	1.4	<0.01	0.07	**1.47** (1.39–1.55)
Heavy vehicle	2630 (9.5%)	1.1	550 (6.3%)	0.7	<0.01	0.05	0.67 (0.61–0.73)
Motorcycle	539 (1.9%)	1.0	56 (0.7%)	0.3	<0.01	0.04	0.33 (0.25–0.44)
**Day period**							
Night	17917 (57.5%)	1.0	4374 (47.3%)	0.9	<0.01	0.09	0.82 (0.80–0.84)
Day	13222 (42.5%)	1.0	4869 (**52.7%**)	1.2	<0.01	0.09	1.24 (1.21–1.27)
**Week period**							
Long Weekend	3952 (12.7%)	1.0	1203 (13.0%)	1.1	0.42	<0.01	1.03 (0.97–1.09)
Weekend	16078 (51.6%)	1.0	4465 (48.3%)	1.0	<0.01	0.03	0.94 (0.91–0.96)
Weekday	11109 (35.7%)	1.0	3575 (38.7%)	1.0	<0.01	0.03	1.08 (1.05–1.12)
**Vacation period**							
Non-vacation	22096 (71.0%)	1.0	6485 (70.2%)	1.0	0.14	<0.01	0.99 (0.97–1.00)
Vacation	9043 (29.0%)	1.0	2758 (29.8%)	1.0	0.14	<0.01	1.03 (0.99–1.07)

* 2-tailed P-value, based on Yates corrected Chi-Square

^ Impact Factor

Bi-variate descriptive analysis was undertaken to analyse the distribution of fatal RTCIs that occurred among the different road user categories (transport mode and sex) in relation to relevant crash related risk factors using the following measures of proportion:

1. **Simple proportion**, calculated for fatal injuries within the categories of the transport mode and sex variables.
2. **Population-based fatality rate**, based on Statistics South Africa mid-year population estimates for 2017, and calculated for males and females to quantify geographical risk by province and municipality.
3. “**Impact factor**”, reflecting the proportion of fatal injuries that occurred among a road user category compared to the proportion of fatal crashes for a respective crash-related risk category, calculated as the quotient between these two proportions. The impact factor provides an indication of the vulnerability conferred upon a road user from a particular category of the crash related variables. In addition, for the temporal risks, impact factors were derived from the proportion of fatal crashes relative to the proportional number of applicable days over the three-year period.
4. **Crash severity**, calculated as a simple ratio of the overall number of fatalities to the total number of crashes for each of the risk categories.

Chi-Square analysis, using the OpenEpi v3.01 software [36] was undertaken to compare the proportions and rates between categories of the transport mode and sex variables in relation to the crash related factors. Fatalities involving riders of motorised 2- and 3-wheelers were considered under the “driver” category rather than as a separate group due to their very small number of cases (1.7% of all fatalities), which would have compromised disaggregated analyses due to small numbers. In addition, given that motorcycle riding in South Africa is generally associated with pastimes among advantaged populations [37], it is conceptually relevant for these cases to be treated as distinct from the remaining categories that reflect disadvantage (passengers and pedestrians), and to allow for an assessment of road user vulnerability from an equity and social justice perspective.

Two-tailed P-values (based on Yates corrected Chi-Square) and risk ratios with 95% confidence intervals are presented. For mode of transport, pedestrian fatalities were compared against a group of all vehicle occupant fatalities (driver and passenger fatalities combined). In view of the large sample sizes of the disaggregated comparison groups, the Cramer’s V statistic was used to provide a measure of effect size. Cramer’s V values range from 0–1 where larger values reflect a stronger relationship between the comparison variables [38]. Values > = 0.1 are generally considered as having practical relevance with values up to < = 0.3 indicative of a “small” effect size [38]. The effect size and other analyses were undertaken using the Statistical Package for Social Sciences (SPSS) version 25. The level of statistical significance was set at p < 0.05 for all relevant analyses.

The above measures and statistics are presented in Tables 1–4 and described further in the results.

**Table 4:** Population-based fatality rates by road user sex, province, and municipality.

	Male			Female			Total			Male vs. female	
	N	Pop.	Rate^	N	Pop.	Rate^	N	Pop.	Rate^	*P* *	Cramer’s V
EC	3607	3147456	38.2	1305	3545488	12.3	4984	6692944	24.8	<0.01	0.01
Local	2693	2195558	40.9	1038	2500133	13.8	3784	4695691	26.9	<0.01	0.01
Metro	914	951898	32.0	267	1045356	8.5	1200	1997253	20.0	<0.01	0.02
FS	2072	1380864	50.0	746	1482142	16.8	2856	2863006	**33.3**	<0.01	0.01
Local	1646	976051	**56.2**	605	1045598	**19.3**	2287	2021649	37.7	<0.01	0.02
Metro	426	404813	35.1	141	436544	10.8	569	841357	22.5	<0.01	0.01
GP	6419	7244846	29.5	1540	7201562	7.1	8037	14446408	**18.5**	<0.01	0.01
Local	1362	956488	**47.5**	378	904932	**13.9**	1759	1861420	31.5	<0.01	0.02
Metro	5057	6288358	26.8	1162	6296630	6.2	6278	12584988	16.6	<0.01	0.01
KZN	5976	5251400	37.9	1779	5784916	10.3	7919	11036317	23.9	<0.01	0.02
Local	3937	3380697	38.8	1253	3879231	10.8	5305	7259929	24.4	<0.01	0.02
Metro	2039	1870703	36.3	526	1905685	9.2	2614	3776388	23.1	<0.01	0.02
LIM	3723	2773381	44.7	1125	3109696	12.1	4893	5883077	27.7	<0.01	0.02
Local	3723	2773381	44.7	1125	3109696	12.1	4893	5883077	27.7	<0.01	0.02
Metro	NA	NA	NA	NA	NA	NA	NA	NA	NA	NA	NA
MP	3451	2187706	52.6	947	2260902	14.0	4473	4448608	**33.5**	<0.01	0.02
Local	3451	2187706	52.6	947	2260902	14.0	4473	4448608	33.5	<0.01	0.02
Metro	NA	NA	NA	NA	NA	NA	NA	NA	NA	NA	NA
NC	897	608760	49.1	284	624289	15.2	1195	1233048	**32.3**	<0.01	0.02
Local	897	608760	49.1	284	624289	15.2	1195	1233048	32.3	<0.01	0.02
Metro	NA	NA	NA	NA	NA	NA	NA	NA	NA	NA	NA
NW	2318	1976285	39.1	740	1921185	12.8	3090	3897470	26.4	<0.01	0.01
Local	2318	1976285	39.1	740	1921185	12.8	3090	3897470	26.4	<0.01	0.01
Metro	NA	NA	NA	NA	NA	NA	NA	NA	NA	NA	NA
WC	2676	3249246	27.5	777	3347733	7.7	3564	6596979	**18.0**	<0.01	0.01
Local	1333	1121655	39.6	432	1163938	12.4	1823	2285593	26.6	<0.01	0.02
Metro	1343	2127591	21.0	345	2183795	5.3	1741	4311386	13.5	<0.01	0.01
**Total Local**	21360	16176581	44.0	6802	17409904	13.0	28609	33586485	28.4	<0.01	0.02
**Total Metro**	9779	11643363	28.0	2441	11868009	6.9	12402	23511372	17.6	<0.01	0.01
**Total National**	31139	27819944	37.3	9243	29277913	10.5	41011	57097857	23.9	<0.01	0.02

*2-tailed P-value

^ Rates based on Statistics South Africa mid-year population estimates for 2017 (Statistical release P0302, https://www.statssa.gov.za/publications/P0302/P03022017.pdf)

# Abbreviations for provinces: EC = Eastern Cape; FS = Free State; GP = Gauteng; KZN = KwaZulu-Natal; LIM = Limpopo; MP = Mpumalanga; NC = Northern Cape; NW = North West; WC = Western Cape

## Results

In explicating road user risk and associated issues of vulnerability and inequity in the South African road traffic environment, the disaggregated number of fatalities in relation to factors pertaining to the characteristics of the crashes are examined for transport modes (Tables 1 and 2) and for males and females (Table 3). In addition, disaggregated population-based fatality rates for males and females to quantify geographical risk by province and municipality are presented in Table 4. Only crash-related factors of interest in differentiating risk between the road user groups are included in the tables. Also not included in the tables are measures of crash severity, however, notable findings are included in the text below. The tables show a comparison in group proportions using Chi-Square, Risk Ratios and Impact Factors while the Cramer’s V statistic reflects the strength of association between the comparison variables (road user group vs. crash characteristic).

Over the 3-year period from 2016–2018, 33 659 fatal crashes were recorded that accounted for 41 011 fatalities. A decreasing trend was observed with the recorded numbers of fatal crashes and fatalities decreasing over the three years by roughly one-tenth, from 11 676 to 10 546 for fatal crashes, and from 14 071 to 12 890 for fatalities. Pedestrians accounted for the largest proportion of fatalities (39.5%), followed by vehicle passengers (33.7%) and vehicle drivers (26.8%). More than three-quarters (77.1%) of the victims were male.

The disaggregated findings for the road user groups are presented below, organised by the relevant crash-related risk factors. Following our focus on issues of vulnerability and inequity, the key focus in presenting the results is on pedestrian and female risk and to a lesser extent, on passengers. As indicated, due to limitations with the RTMC data, passenger cases could not be disaggregated further by vehicle type to explicitly examine vulnerability of public transport passengers, although some relevant content is presented to provide an indication of public transport vulnerability.

### Crash cause

Overall, the cause of crash was most often attributed to driver factors (42.2%) followed by pedestrian factors (35.1%). Specifically, the largest attribution to driver factors was speeding (22.1% of all crashes) and to pedestrian factors was jaywalking (34.1% of all crashes). Fatal crashes attributed to driver factors showed a marginally higher crash severity (1.4) compared to fatal crashes attributed to vehicle and road factors (1.3 each), indicating that on average, each fatal crash resulted in 1.4 fatalities in the case of those attributed to driver factors and 1.3 fatalities in the case of those attributed to vehicle and road factors.

The proportional distribution in the crash causes was generally similar for females and males however, of note, was the relatively larger impact factor for females involved in fatal crashes attributed to vehicle factors (1.5 for females vs. 1.0 for males), indicating that females were 1.5 times more likely to die in such crashes (Table 3).

### Crash type

Overall, crashes between drivers and pedestrians accounted for the largest proportion of the total fatal crashes (42.5%).

The proportional distribution and impact factors between females and males were generally similar across the categories of crash type. Females, however, had a marginally larger proportion of fatal crashes that occurred between two or more vehicles as well as a relatively higher impact factor (1.4 vs. 1.2 for males), indicating that females were 1.4 times more likely to die in crashes between two or more vehicles whereas males were 1.2 times more likely to die in such crashes (Table 3).

### Vehicle type

Light vehicles were involved in more than three-quarters (77.1%) of the total fatal crashes. Among the different types of vehicles, the largest crash severity was found for public transport followed by heavy vehicles (1.4 and 1.3, respectively), indicating that on average, each fatal crash resulted in 1.4 fatalities in the case of public transport vehicles and 1.3 fatalities in the case of heavy vehicles. The relatively higher crash severity for public transport vehicles is consistent with their relatively higher passenger capacities while crashes with heavy transport vehicles were found to be associated with more vehicles per crash (average 1.2 vs. 0.97 for public transport vehicles).

Both pedestrians and passengers were disproportionately vulnerable in fatal crashes involving public transport vehicles, observed to be 1.4 and 1.3 times more likely respectively to die in crashes involving public transport vehicles. Impact factors were higher for buses than for minibuses for both pedestrians (1.5 vs 1.4, respectively) and for passengers (1.5 vs 1.3, respectively). Impact factors for other modes of transport did not exceed 1.0. In terms of relative risk, pedestrians had a significant 1.5 times higher fatality risk than vehicle occupants in crashes that involved public transport vehicles (Table 1). The relative risk was marginally higher for crashes involving minibuses (1.5) as compared to crashes involving buses (1.4).

Females were involved in a larger proportion of fatal crashes that involved public transport vehicles (18% vs. 12% for males) and with a markedly higher impact factor (1.4 vs 1.0 for males), indicating that females were 1.4 times more likely to die in crashes involving public transport vehicles. The impact factor was higher for buses than for minibuses (1.7 vs 1.4, respectively). The relative risk was also higher for crashes involving buses (1.7) as compared to crashes involving minibuses (1.4).

### Temporal characteristics

Most fatal crashes occurred at night (55.3%), over weekends (62.7%), and during non-vacation periods (71.2%). Relative to the proportional number of days in a year for these temporal periods, impact factors over the 3-year period were 1.5 for long weekends (8.2%), 2.1 for regular weekends (23.6%), and 1.0 for vacation periods (28.8%). That is, fatal crashes were twice as likely to occur over regular weekends, 1.5 times more likely to occur over long weekends, with no differences for vacation versus non-vacation periods.

The impact factors for the road user groups in relation to all temporal factors were negligible with none exceeding 1.2. The proportional distribution and relative risks between the road user groups were also similar (Table 1).

Females had 24% more fatal crashes than males that occurred during the day, along with a slightly higher impact factor of 1.2 (vs 1.0 for males), indicating that females were 1.2 times more likely to die in in such crashes. For day of week and vacation periods, impact factors and group differences were unremarkable (Table 3).

### Area-level characteristics

#### Road type and road surface condition

With road-related factors, 7.2% of fatal crashes occurred on unpaved roads (including dirt and gravel roads) and 5.2% occurred under wet conditions. In terms of road user groups, most findings were unremarkable with an exception for a significant 1.7 times higher fatality risk found for pedestrians occurring on dirt roads as compared to that for vehicle occupants (Table 3).

#### Province and municipality

The highest fatality rates per 100 000 population were found for relatively rural provinces of Mpumalanga, Free State and Northern Cape (33.5, 33.3 and 32.3, respectively) (Table 4). The highest crash severity was also found for the relatively rural provinces of Eastern Cape and Free State (1.3 each), indicating that on average, each fatal crash occurring in these provinces resulted in 1.3 fatalities. The lowest fatality rates per 100 000 were found for the Western Cape followed by Gauteng (18.0 and 18.5, respectively). The Western Cape and Gauteng are also the only provinces with larger metropolitan than local municipality populations (Table 4).

More than two-thirds (67.1%) of fatal crashes occurred in local municipality areas as compared to metropolitan municipalities. In relation to the national fatality rate computed at 23.9 per 100 000 population, the rate for local municipalities at 28.4 was roughly 1.5 times that of metropolitan municipalities at 17.6 (Table 4). Overall, fatal crashes in local municipalities were found to have a higher crash severity than metropolitan municipalities (1.3 vs 1.1). That is, on average, each fatal crash occurring in a local municipality resulted in 1.3 fatalities, whereas each fatal crash in a metropolitan municipality resulted in 1.1 fatalities.

#### Mode of transport by province and municipality

The largest proportion of pedestrian fatalities was found for Gauteng and KwaZulu-Natal (24.2% and 24.1%, respectively). KwaZulu-Natal also recorded the largest proportion of passenger fatalities (16.9%), followed by Limpopo (15.2%) (Table 2).

Pedestrians were particularly vulnerable in metropolitan municipalities, shown to be 1.3 times more likely to die in crashes occurring in metropolitan municipality areas (versus 0.9 in local municipality areas). That is, pedestrians were 1.4 times more likely to die in metropolitan as compared to local municipality areas. Notable impact factors by province were found for pedestrian fatalities in metropolitan municipality areas in KwaZulu-Natal (1.4), and for passenger fatalities in local municipality areas of Northern Cape and Limpopo (1.5 and 1.4, respectively) (Table 2).

In terms of risk and based on the risk ratios between pedestrians and vehicle occupants, an overall 93% higher proportion of pedestrian fatalities was found for metropolitan municipality areas and a 36% higher proportion of vehicle occupant fatalities was found in local municipality areas, both being statistically significant and also shown to have the largest effect sizes of 0.22 each. Specifically, significantly large relative risks were found for pedestrians in relation to vehicle occupants in metropolitan municipalities of KwaZulu-Natal followed by Western Cape, Gauteng and Eastern Cape (RR 2.9, 2.4, 1.7 and 1.5, respectively). The effect sizes for KwaZulu-Natal and Gauteng were notable with Cramer’s V values of 0.14 and 0.12, respectively (Table 2). The generally low Cramer’s V values found across the variables are discussed in the following section.

#### Road user sex by province and municipality

The overall fatality rate per 100 000 population for males at 37.3 was 3.5 times higher than that for females at 10.5, varying by a factor of 4.1 in metropolitan municipalities and 3.4 in local municipalities. The fatality rates for both males and females were higher in local municipality areas than in metropolitan municipality areas for all provinces (where applicable). Specifically, for both males and females, the highest fatality rates per 100 000 population were found in local municipality areas of the Free State (56.2 and 19.3, respectively) followed by local municipality areas of Gauteng (47.5 and 13.9, respectively) (Table 4).

## Discussion

This research has shown clear differentials in the risk of RTCIs, both among and between road users (using impact factors and relative risk, respectively), and for categories of transport mode and sex of road user. The findings, disaggregated in the first instance by vulnerable road users, provide a perspective on vulnerability from a human biomechanical vulnerability perspective. Disaggregated further by characteristics of the crashes, key findings include higher pedestrian risk in relation to public transport vehicles and with area-level influences such as the nature of roads or extent of urbanity; higher passenger risk in relation to public transport vehicles and with influences of rurality; and higher female road user risk in relation to public transport vehicles. The findings provide important perspectives to contextual influences from the transport environment in relation to the differentiated findings for road users and to the disproportionately large burden of RTCIs in the country in general. Based on our findings, we bring attention below to two priority issues: pedestrians in relation to matters around socio-economic and area-level deprivation, and public transport passenger risk in relation to female road users.

### Pedestrian vulnerability

Pedestrians accounted for the largest proportion of fatalities (39.5%) and based on the impact factor measure, these road users were 2.1 times more likely to die in crashes involving public transport vehicles, 1.7 times more likely in crashes occurring on dirt roads and 1.3 times more likely in crashes occurring in metropolitan municipality areas. The relatively higher vulnerability of pedestrians in crashes occurring in metropolitan municipalities is consistent with local and international research [7, 39, 40]. Specifically, in the South African setting, a general increase in population-based fatality rates with increasing levels of urbanisation was demonstrated for pedestrians, measured using several area- and population-based measures of rurality [7]. In addition, high rates of serious injuries and crash severity (a ratio index of both fatal and serious injuries to number of collisions) were shown to be strongly related to area level deprivation proxied by the percentage population living in informal shack settlements [20]. Furthermore, post-apartheid large-scale unplanned migration to cities has also contributed to a general “urbanisation of poverty” [41], commonly associated with a multitude of social and area-level deprivations including informal shack settlements [4, 20]. Other challenges to pedestrians in deprived settings, include the relatively higher travel exposure and inadequate safety provisioning with pedestrian-related infrastructure. The general preponderance of informal commercial activities specific to deprived areas in urban settings may also be an important contributor to the higher risk exposure for pedestrian-vehicle incidents.

### Public transport passenger vulnerability

Vehicle passengers accounted for one-third of all road traffic deaths (33.7%) and based on the impact factor measure, they were 1.3 times more likely to die in crashes involving public transport vehicles (1.5 times for buses and 1.3 times for minibuses), and 1.2 times more likely in crashes occurring in local municipality areas. Local municipalities, generally characterized by higher proportions of rural areas, also exhibited higher crash severities than relatively more urbanised metropolitan municipalities, with fatality-to-crash ratios of 1.3 and 1.1 respectively. As with pedestrian injury risk, the added travel and risk exposure to public transport passengers is also associated with the legacy of apartheid spatial planning. The generally larger extent of RTCIs in rural areas has been demonstrated in local and international research [4, 40, 41] and may be attributed to higher travel exposure from longer distances travelled, faster speeds for such longer journeys along with relatively unsafe roads that result in more severe collisions and injuries, and to poorer injury outcomes owing to sub-optimal access to quality pre-hospital and advanced in-hospital trauma care.

### Female vulnerability

Like in most other country settings, males are overrepresented in the mortality burden of RTCIs with aggregated data for the South African context showing more than three-quarters of the victims of fatal road traffic crashes to be male. Whilst the mortality risk for men is greatest, the greater burden of vulnerability is transferred by this mortality onto surviving women and children, including family instability and financial hardship. This financial hardship is of particular importance in the South African context where males dominate as primary income earners especially in disadvantaged rural settings [26] where socio-economic disadvantage are exacerbated.

Notwithstanding the overall higher absolute risk for males, this research shows that females experience greater relative risk and vulnerability with public transport. Specifically, females were 1.4 times more likely to die in crashes involving public transport vehicles, with a much higher likelihood for buses than for minibuses (IF 1.7 and 1.4, respectively). From a relative risk perspective, females were also more likely than males to die in crashes involving both buses and minibuses (RR 1.7 and 1.4, respectively).

Women have been shown to use public transport more often than men in general [22]. The relatively higher risk to females in crashes involving buses is also emphasised with findings from the NHTS [22] reflecting higher exposure with minibus taxis (rather than buses) in lieu of minibus taxi travel being the dominant mode of transport for females. Research on the vulnerability of women in using bus and minibus transport is scant, hence the reasons for the relatively higher risk with buses compared to minibuses is not known. However, findings from research on female experiences with rail transport such as greater travel exposure and experiences of unsafety and insecurity [27] may also apply in varying degrees to bus and minibus passenger transport. Specific to bus and minibus exposure, the experience of some specific contextual risks may also be more pronounced for females such as with public transport operators (especially informal minibuses) collecting and dropping of passengers in unsafe areas, along with users needing to negotiate such complexities and potential conflicts. This experience would be exacerbated given that women are more likely to travel with children and/or with loads [42].

### Policy and programming recommendations

This research highlights the need for prioritising specific measures to enhance the safe mobility of vulnerable road users, specifically pedestrians, passengers of public transport and female road users.

In terms of traffic-related interventions, our findings provide support for prioritising the protection of pedestrians through passive infrastructural upgrades such as safe walkways and crossings, and the stricter monitoring and regulation of unsafe behaviours and practices by public transport operators. A further short- to medium-term strategy is for optimising the collection, analysis and systematic reporting of sex-disaggregated data to allow for identifying differentiated risks and for informing sex-sensitive policies and strategies. Opportunity in this regard lies with optimising the design of the country’s national household and travel surveys.

Medium- to longer-term strategies should seek to prioritise sex and equity integration in traffic, transport and broader development policies. Strategic traffic-related priorities based on our findings would include intensifying efforts at regulating the informal taxi industry, provision of safe and reliable public transport that is sensitive to the vulnerability of females, and remedial urban planning strategies to help correct for high levels of travel-related exposure among vulnerable road users in disadvantaged settings.

## Limitations and opportunities for further research

Whilst this study has yielded valuable findings in defining the risk differentials amongst vulnerable road users, further research is needed to optimise the analytical variables used and to explicate findings beyond the crash related factors considered in this research.

In this study, we used population-based rates as an indicator of risk exposure. While population-based rates are helpful in quantifying the public health burden for aggregated road traffic injury outcomes, its use is limited in differentiating the risk faced by individual or cohort road users. Better suited are mobility or travel exposure metrics such as time or distance of travel or the number of trips undertaken for a transport mode of interest. Data on distance and time of travel was also not available, hence it was not possible for us to calculate and compare exposure-based risk between the road user categories. Opportunity lies with integrating such metrics within the country’s NHTS with the inclusion of data on age in order that issues around age equity may also be investigated in relation to the exposure-based metrics.

The Cramer’s V values found from this study did not exceed 0.2. Based on the Cohen’s thresholds, the strength of association between our comparison variables may therefore be regarded as weak. However, these commonly adopted traditional thresholds may not be entirely appropriate in view of growing criticisms that these generic thresholds, based principally upon a qualitative impression, do not give due consideration for the disciplinary context and type of data being used [43, 44]. For example, based on a review of 708 meta-analytically derived correlations from the psychology literature, the average effect size was determined at 0.19 with <3% of correlations found to be as large as 0.50 [43]. The generally low Cramer’s V values from this research are also a reflection on the multiple and interacting influences on RTCIs in general, and more specifically on other influences not considered in this research. Hence, overall estimates of road user risk may also be improved through the adoption of stronger study designs and a wider range of explanatory effects across the spectrum of the proposed conceptual framework including those relating to travel exposure, travel patterns and risky behaviours. More specifically, opportunity lies with obtaining insights to better understand the vulnerability of female passengers in bus and minibus transport, as has been undertaken for females using rail transport [27], and with disentangling the relative and interacting effects across the proposed 3-dimension “User-System-Context” on vulnerability using robust multivariate analytic modelling techniques.

## Conclusion

The risk differentials shown in this research for vulnerable road user groups such as pedestrians, public transport passengers and female road users provides important perspectives for addressing the equitable access to safe mobility for these road users in the country. Based on our findings, we draw attention to the need for prioritization of the range of important structural effects relating to social and area level deprivation. We further make the case for an explicit focus on these deprivation-related structural effects within a 3-dimension “User-System-Context” proximal-to-distal continuum framework to conceptualise and address vulnerability in the transport system in its totality. In so doing, we add to the imperative for an increased focus on the root causes of vulnerability pertaining to the transport system and structural influences as opposed to an inordinate focus on road users and their vulnerabilities in efforts to reduce the burden of RTCIs.
